# A Unified Approach to Linking Experimental, Statistical and Computational Analysis of Spike Train Data

**DOI:** 10.1371/journal.pone.0085269

**Published:** 2014-01-17

**Authors:** Liang Meng, Mark A. Kramer, Steven J. Middleton, Miles A. Whittington, Uri T. Eden

**Affiliations:** 1 Department of Mathematics and Statistics, Boston University, Boston, Massachusetts, United States of America; 2 Hull York Medical School, York University, York, United Kingdom; McGill University, Canada

## Abstract

A fundamental issue in neuroscience is how to identify the multiple biophysical mechanisms through which neurons generate observed patterns of spiking activity. In previous work, we proposed a method for linking observed patterns of spiking activity to specific biophysical mechanisms based on a state space modeling framework and a sequential Monte Carlo, or particle filter, estimation algorithm. We have shown, in simulation, that this approach is able to identify a space of simple biophysical models that were consistent with observed spiking data (and included the model that generated the data), but have yet to demonstrate the application of the method to identify realistic currents from real spike train data. Here, we apply the particle filter to spiking data recorded from rat layer V cortical neurons, and correctly identify the dynamics of an slow, intrinsic current. The underlying intrinsic current is successfully identified in four distinct neurons, even though the cells exhibit two distinct classes of spiking activity: regular spiking and bursting. This approach – linking statistical, computational, and experimental neuroscience – provides an effective technique to constrain detailed biophysical models to specific mechanisms consistent with observed spike train data.

## Introduction

Accurate representation of real world phenomena typically requires detailed computational models, which must be constrained by extensive, carefully measured data sets. This issue is particularly relevant in contemporary neuroscience research, in which both detailed computational models and large data sets are now common for the activity of an individual neuron. Diverse spiking patterns result from many interacting biophysical mechanisms, including ion channels intrinsic to the neuron and electrical and chemical signaling between neurons [1]. Understanding the relationships between observed spiking patterns and their generative mechanisms remains an active research area with many sophisticated approaches, both computational [2] and experimental [3]. The diversity of mechanisms responsible for spike generation, and the nonlinear interactions between these mechanisms, makes linking observed spike activity to specific mechanisms a challenging task. Specifically, given an observed spike pattern what, if anything, can we conclude about the underlying mechanisms?

Various approaches exist to address this question. In experiments, the proposed mechanisms of spike pattern generation can be tested directly through pharmacological manipulations, although this procedure can be time consuming, expensive, and inexact (e.g., due to the nonspecific impacts of some drugs). In computational modeling of an observed spike pattern, a common approach is hand-tuning, which requires first proposing a computational model (e.g., the Hodgkin-Huxley model neuron [4]) and then adjusting the model parameters until a qualitative match with the observed spike pattern is found [5]. This approach is time consuming, requires extensive training and expertise, and is restrictive; often only a single parameter configuration is determined, and the full parameter space capable of generating the observed spike activity is left unknown [6]–[8]. An alternative approach is to develop simplified statistical models that describe empirical features of the spiking [9], [10]. These models are readily constrained by the data, but cannot be directly connected to physiological mechanisms.

Recent approaches to overcome the limitations of hand-tuning include brute-force simulations over broad intervals of parameter space [5], [11], and estimation of model parameters directly from the observed neuronal voltage activity [12]–[17], or the observed spike pattern [18]–[20]. We recently proposed a new approach to quantitative parameter estimation from neuronal spike patterns [21]. This parameter estimation framework combined a conductance based biophysical model of neuron voltage activity (i.e., a Hodgkin-Huxley type model neuron) with point process statistical theory to estimate model components directly from an observed spike train. The estimation algorithm utilizes an established statistical procedure, known as particle filtering or sequential Monte Carlo (SMC), which has been increasingly applied to characterize the dynamical features of detailed stochastic computational models with many unknown parameters and variables. Compared to hand-tuning, the particle filter procedure allows a principled exploration of a parameter space and identification of multiple parameter sets consistent with the observed activity [21].

Here we apply this estimation procedure to spike time data collected from living neurons recorded *in vitro*. Specifically we analyze the spike time data recorded from rat layer V intrinsically bursting (IB) neurons. We choose the IB neuron because it possesses an intrinsic current – the muscarinic receptor suppressed current or M-current (e.g., [22]–[25]). A detailed experimental and computational modeling study has shown that the M-current is the primary driver of the rhythmic activity in the spike time data analyzed here; for the experimental characterization of this current, including pharmacological manipulations, please see [26]. Then, given only the spike time data recorded from an IB neuron, we estimate parameters in a generic Hodgkin-Huxley type computational model. We construct this model to possess the standard intrinsic currents necessary for spike generation, plus an additional “mystery” current with unconstrained characteristics. In what follows, we show that the particle filter framework successfully constrains the parameters of the mystery current in ways consistent with the expected characteristics of an M-current. In doing so, we will show how the same intrinsic current can support different types of spiking behavior (namely, rapid spiking and bursting) dependent upon the interplay of two model parameters related to the overall excitability and strength of the mystery current.

We begin by applying a standard point process analysis paradigm, and construct both descriptive statistics and a simple statistical model to provide an initial characterization of the spike train data. We then propose a computational model of these data: a Hodgkin-Huxley type neuron with an additional, generic intrinsic current. To estimate parameters of this unknown current, we implement a particle filter framework, and show that this approach successfully identifies the features of a current consistent with the known M-current in the IB cells. In this way, by linking techniques from statistical and computational neuroscience, we analyze experimental spike train data to gain insight into the biophysical mechanisms driving the observed activity.

## Results

The goal of this paper is to associate with an observed spike train a specific biophysical mechanism through a combination of statistical techniques and computational modeling. To start, we first consider visualization and descriptive statistics applied to four IB cells. These traditional analyses illustrate the spiking patterns of each cell, and separate the observed activity into two classes of behavior: rapid spiking and bursting. We then develop statistical models of each cell to further illustrate the characteristics of the observed behavior. Finally, we employ a sequential Monte Carlo (SMC) or particle filter method to estimate parameters in a computational model and thereby identify biophysical mechanisms consistent with the observed spike trains. Through this technique, we show that the same intrinsic current – a slow, depolarization activated, hyperpolarizing current, consistent with a known intrinsic current of the IB cells – supports the two distinct dynamic regimes of activity.

### Visualization and descriptive statistics

We begin with visualization of the spike train data using descriptive statistical methods [27]. Figure 1.1 shows an example spike train from cell 1 (Figure 1.1A), the histogram of its inter-spike intervals (ISI) for the entire 60 seconds of observation (Figure 1.1B), and the spectrum of the discretized spike train data with sampling interval 1 ms (Figure 1.1C). Figure 1.2–1.4 present the same analyses for the other 3 IB cells. From visual inspection of Figure 1.1A, we conclude that the most common inter-spike interval for cell 1 is approximately 90 ms. This is verified in Figure 1.1B: there appears a single mode of inter-spike intervals near 90 ms. A strong refractoriness is also obvious as we observe no inter-spike intervals below 40 ms. The broad peak centered near 10 Hz and slow recovery of the spectrum to the average firing rate in Figure 1.1C corroborate these results. We conclude that the spiking activity from cell 1 exhibits rhythmic behavior near 10 Hz, and a long refractory period. Cell 2 exhibits a similar pattern of spiking activity to cell 1 (Figure 1.2 A–C). However, unlike cells 1 and 2, the spiking activity of cell 3 occurs at a higher rate with two modes of frequent inter-spike intervals: one is small, around 10 ms and another is about 4 times bigger, around 45 ms; both are apparent in the ISI histogram (Figure 1.3 A, B). The refractory period is made clear by the dramatic drop of spiking when the inter-spike interval is below 10 ms. The sharp peak around 25 Hz (the beta2 band) and broad peak around 100 Hz of the spectrum in Figure 1.3C support the conclusion of two rhythmic spiking modes existing in cell 3. Cell 4 (Figure 1.4 A–C) has a similar spiking pattern to cell 3, namely fast rhythmic activity and bimodality of the ISI distribution. Thus we find two distinct activity regimes in the same subclass of neuron: (1) spike behavior with slight rhythmicity in the beta2 range without clear bursting behavior (cells 1 and 2), and (2) spike behavior in which spike times are organized into beta2 frequency burst discharges (cells 3 and 4).

**Figure 1:**
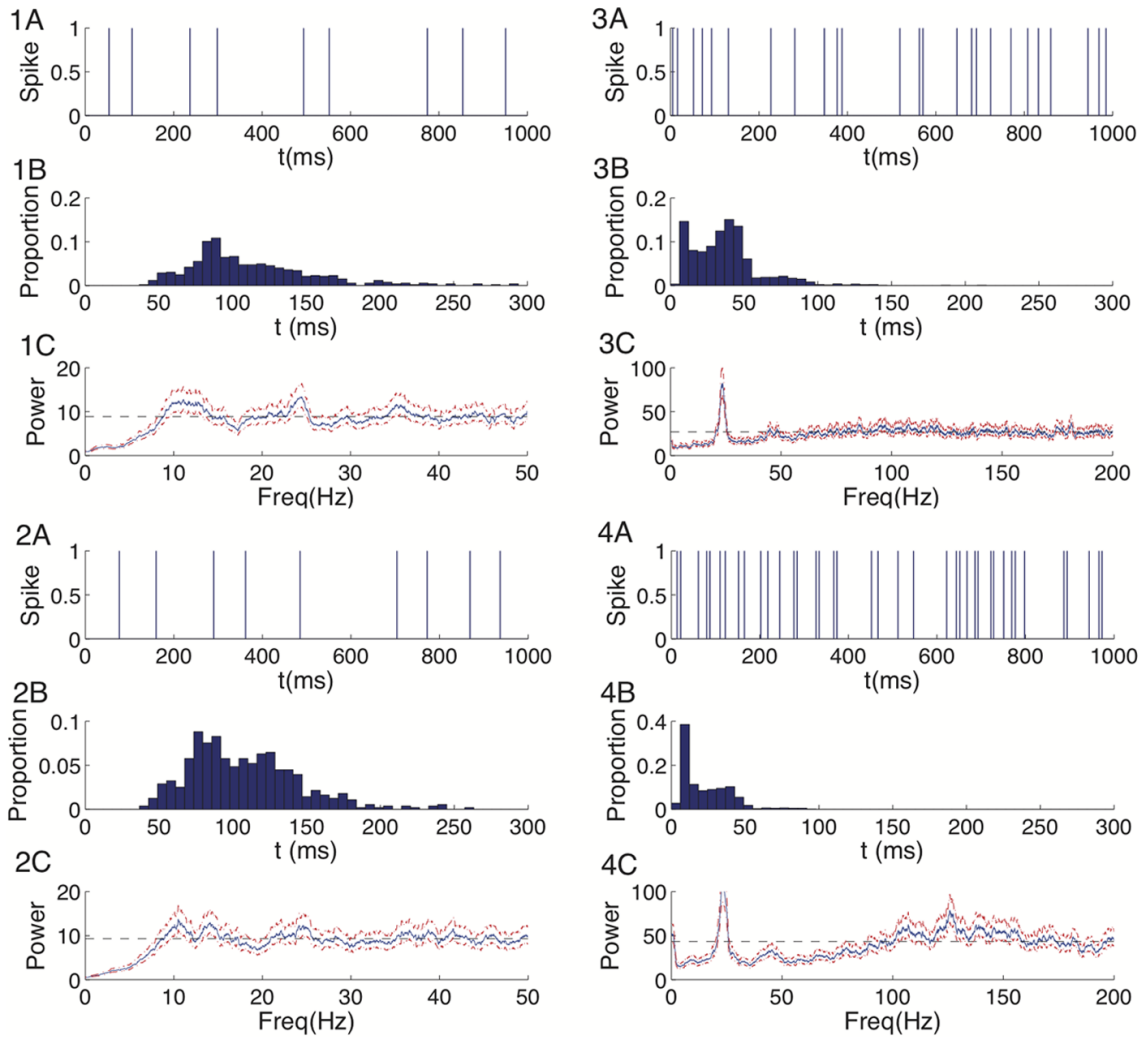
Visualizations and descriptive statistics of the observed spike times suggest two modes of behavior in the same cell class. **1A–4A**: 1 second interval of spike train data for each of the four cells considered. **1B–4B**: The histogram of inter-spike intervals for cells 1–4. **1C–4C**: The spectrum of cells 1–4 (solid blue line) and its 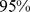 confidence intervals (dashed red lines). The dashed black line indicates the estimate of overall spiking rate 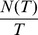 where 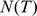 represents the total number of spikes up to time 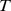.

### Statistical modeling

**Figure 2:**
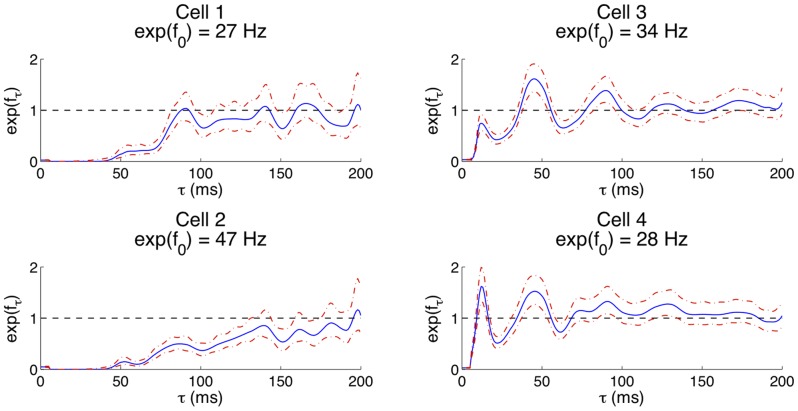
Statistical models of the spiking activity suggest two regimes of behavior. The four subfigures show the fitted values of the history dependence of the firing rate 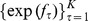 and the 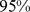 confidence intervals for cells 1–4. The blue lines represent estimates of the history components. The dot-dashed red line indicates the point-wise 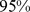 confidence intervals of each estimate.

In this section we develop statistical models – relating the spike rate to past spiking activity – for the four IB cells. The estimation result for the two components of the statistical model (Eq. 1) are shown for all four cells in Figure 2. The 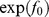 term represents the constant background firing rate, and the 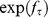 term represents the modulation of background firing rate due to the past spiking of the cell at lag 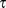. We note that 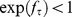 implies a reduction from the background firing rate at lag 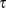, 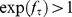 implies an increase above the background firing rate at lag 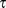, and 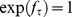 means no influence on the background firing rate at lag 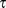. For cells 1 and 2, the modulation of background firing rate is highly reduced until about 50 ms after a spike and then gradually recovers to the background firing rate (i.e., approaches 1). For cell 3 and 4, refractoriness also exists but is shorter than that observed for cells 1 and 2. As the spiking activity approaches 1 (i.e., no modulation), peaks and troughs appear. The first peak occurs around 10 ms after a spike, and implies increased probability of a spike after a 10 ms delay compared to adjacent time lags such as 6 ms and 20 ms. After 40 ms, another peak occurs that exceeds the background spiking rate; during this period the cell again tends to generate more spikes than usual. Following this peak is a trough below the background firing rate near 65 ms, which implies another period of reduced spike probability. After this trough, the spiking rate gradually approaches the background firing rate, which implies minimal effect of history beyond 100 ms on the current firing rate. These statistical modeling results support the observation of at least two different regimes of dynamic activity produced by this cell type.

### Biophysical modeling

**Figure 3:**
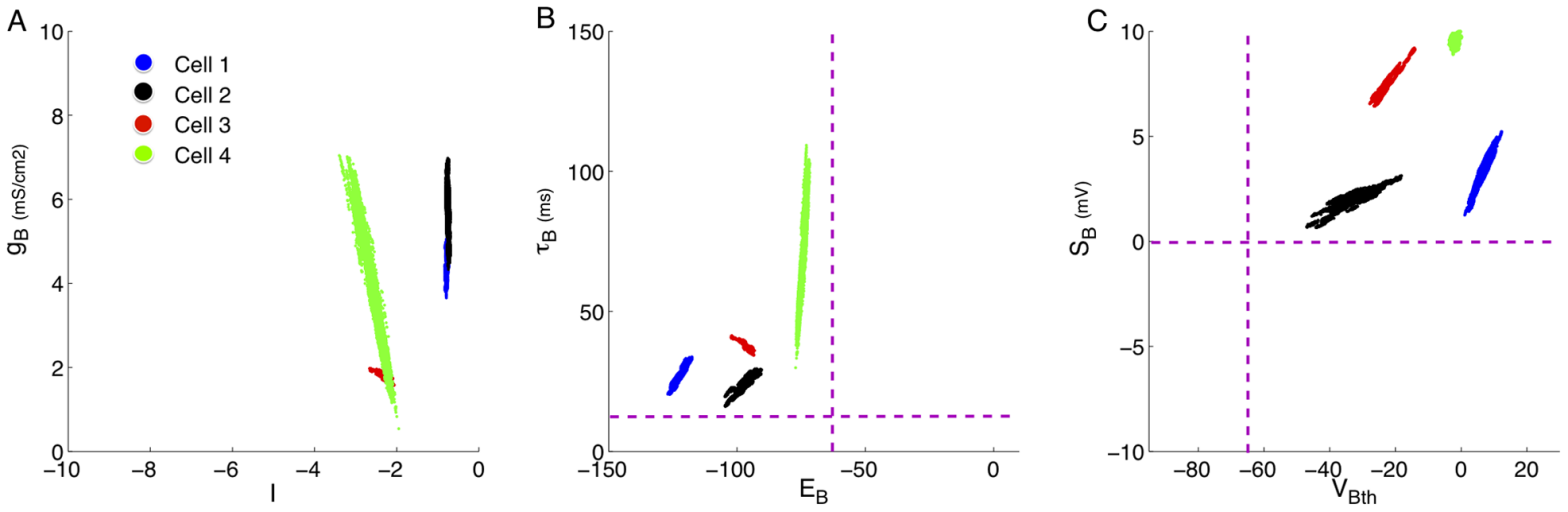
Locations of converged particles for the mystery current model. **A–C**: The blue, black, red, and green dots indicate converged particles for cells 1–4 respectively. **B**: The horizontal dashed line indicates 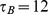 and the vertical dashed line indicates the resting potential −65 mV. **C**: The horizontal dashed line indicates 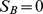 and the vertical dashed line indicates resting potential −65 mV. The three coordinate spaces for each data set span the initial parameter value space in the estimation procedure.

The descriptive analysis and statistical modeling suggest specific features of the observed spike train data, namely long refractory periods for cells 1 and 2, and multiple time courses of spiking for cells 3 and 4. To address the underlying mechanisms supporting these activities, we develop a biophysical model of Hodgkin-Huxley type and search for a common intrinsic current that can support both types of observed activity. The SMC method is applied to estimate the five unknown parameters of the “mystery current” in the constructed model 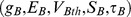, and the overall excitability of the cell 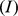. The locations of converged parameter estimates for each cell are shown in Figure 3. We note that for all the cells, the parameter estimates converge to a small region of parameter space. Differences in the sizes of these regions might be attributed to differences in the observation times of the cells (60 seconds for cells 1–3, 30 s for cell 4) or to differences in the information content in the spiking activity about specific parameters or dynamic variables.

From the converged parameter estimates, the biophysical properties of the mystery current can be ascertained by comparing the estimates of 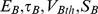. The estimates of these parameters are listed in Table 1. For each cell and parameter to be estimated, the first number indicates the mean of the converged particle values and the second number, in parentheses, indicates the standard deviation of the particle values. In all four cases, we find that the expected 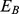 is negative and less than resting potential −65 mV; this suggests that the mystery current acts to hyperpolarize the voltage. 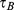 is large compared to the time scale of the standard Hodgkin-Huxley currents which possess timescale on the order of a few milliseconds. These results suggest that the mystery current exhibits slow dynamics. 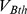 is well above the resting potential of the neuron, and 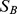 is positive which suggests that the mystery current is depolarization activated. If the constructed biophysical model were completely accurate in representing the IB cells, we would expect overlapping estimates of the characteristic parameters of the “mystery current”, such as 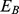 and 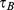. However, we do not find overlap for these parameter estimates. This may suggest that the biophysical model is insufficient to capture all features of the observed spike times. This is of course reasonable: the biophysical model consists only of a single compartment with three intrinsic currents. We might improve upon these models by adding multiple compartments, or additional known currents or dynamics. However, since we know that these simplified or more advanced models are incomplete, we do not interpret the parameter estimates as the actual biophysical values corresponding to these currents. Instead, they provide insight into the types and features of current necessary to produce the observed spiking within the selected class of biophysical models.

**Table 1:** Estimates of unknown model parameters.

	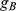	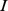	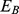	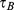	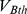	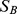
Cell 1	4.4 (0.2)	−0.8 (0.02)	−122 (1.8)	27 (2.6)	6.7 (2.1)	3.3 (0.8)
Cell 2	5.8 (0.5)	−0.8 (0.02)	−97 (2.6)	24 (2.4)	−33 (4.9)	1.9 (0.4)
Cell 3	1.8 (0.1)	−2.3 (0.1)	−96 (2.0)	37 (1.4)	−23 (2.9)	7.4 (0.6)
Cell 4	3.5 (1.3)	−2.5 (0.2)	−74.8 (1.2)	64 (15.0)	−2.2 (0.7)	9.6 (0.1)

Our results suggest that the mystery current for the proposed model would need to be a slow, depolarization activated, hyperpolarizing current in all four cells. This is consistent with the known slow current in these IB cells, a muscarine receptor suppressed potassium current or M-current [26]. We note that the initial assumptions regarding the mystery current are weak, and that other potential currents with different dynamics are attainable. In fact, many other types of currents – fast and slow, depolarization activated and inactivated, hyperpolarizing and depolarizing – are represented in the initial particle values. However, the estimation procedure eliminates these nonconforming particles and reveals in all four cells the characteristics of an M-current. This result suggests that the same type of current species could be responsible for the observed activity in all four cells, even though these cells exhibit very different spiking characteristics (e.g., compare their inter-spike interval distributions in Figure 1). The distinct spiking characteristics may result from the different drive currents 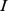 and strengths of the “mystery current” 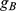. For the cells with similar spiking activity (cells 1 and 2, or cells 3 and 4), the particle clouds of these two parameters are similar and even overlap. However, for the differently spiking cells, such as cells 1 and 3 or cells 2 and 4, the particle clouds of these two parameter estimates remain separate. The stronger expected drive and smaller expected maximum conductance of the “mystery current” for cells 3 and 4 also explains the faster rhythm of spiking in these two cells compared to cells 1 and 2. Our proposed method is not only able to estimate the model parameters but also identifies the characteristics of a mystery current whose specific biophysical mechanisms support the observed activity.

**Figure 4:**
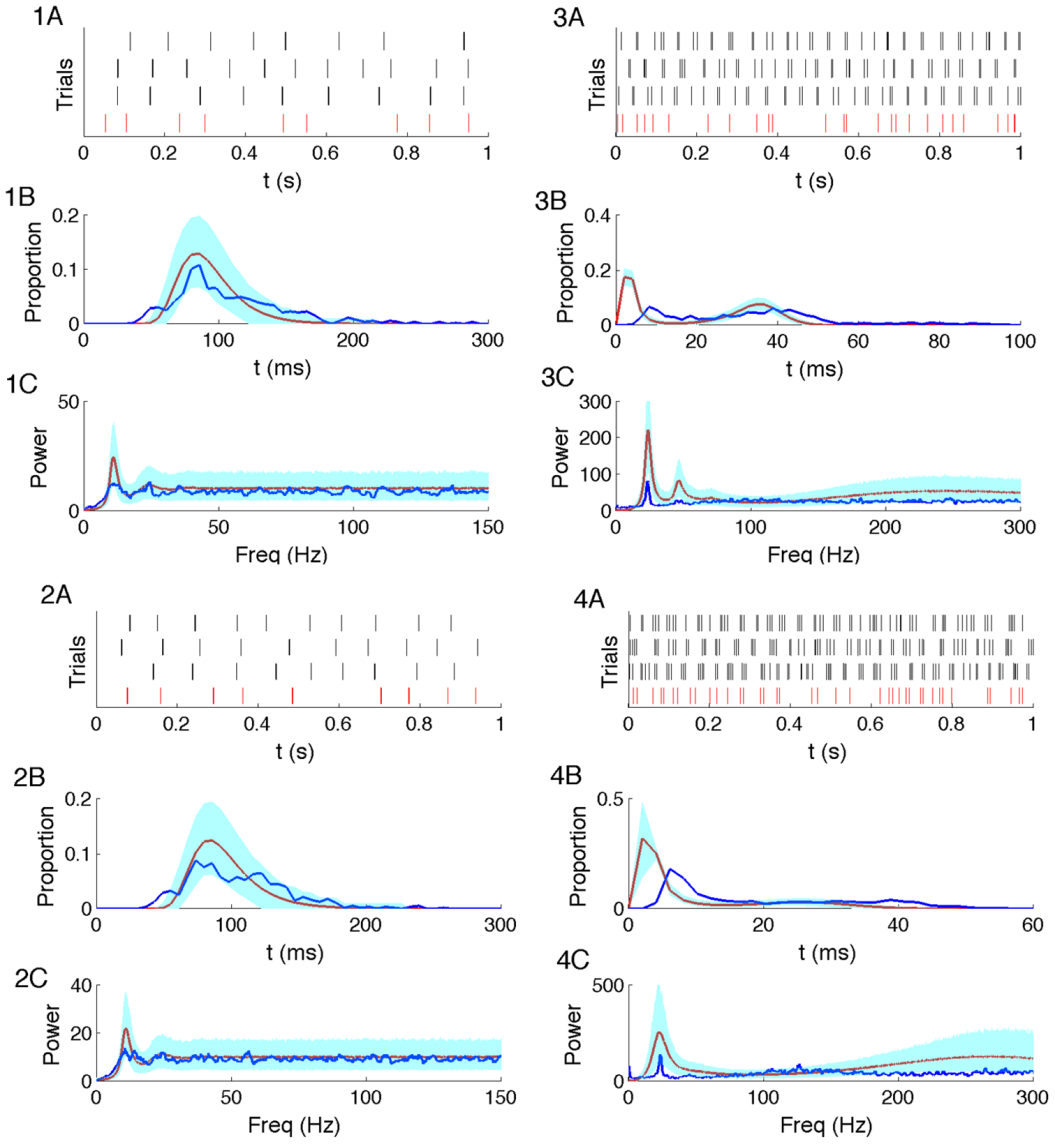
Comparisons between the observed and simulated spike features. **1A–4A**: Four spike trains are shown in this panel. The bottom row (red) represents the observed spike times of cells 1–4 for 1 s of data. The other three rows (black) represent the simulated spike trains from 3 converged particles of cells 1–4. **1B–4B**: The blue line is the ISI histogram of cells 1–4. The red line is the average histogram over all the converged particles of cells 1–4. The cyan band indicates the 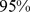 confidence intervals of the average histogram. **1C–4C**: The blue line represents the spectrum estimate of cells 1–4. The red line is the average spectrum estimate over all converged particles of cells 1–4. The cyan band indicates the 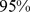 confidence intervals of the average spectrum estimate.

To evaluate the estimation results, we simulate spike times using the converged parameter estimates in the biophysical model (Eq. 2), and compare the descriptive statistics (as in Figure 1) of the simulated spike times to those of the observed spike times as shown in Figure 4. Using the parameter estimates for cells 1 and 2, the simulated spike trains produce tonic spiking activity consistent with the observed spike trains (shown in Figure 4.1A and 4.2A). The average ISI histogram over all the particles (in Figure 4.1B and 4.2B) is unimodal and the peak is near 90 ms, which is again consistent with the real ISI distribution. In addition, the histogram of the observed ISIs falls within the 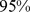 confidence intervals of the simulated ISIs for most times. Finally, the average spectrum of the simulated spike trains over all particles (in Figure 4.1C and 4.2C) has a peak around 10 Hz, consistent with the spectrum of the observed spike trains. At most frequencies the spectrum of the observed spike trains lie within the 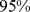 confidence intervals of the simulated spectrum.

Using the parameter estimates for cells 3 and 4, the simulated spike trains (in Figure 4.3A and 4.4A) all show bursting activity, which is consistent with the observed spike trains. The simulated ISI histogram (in Figure 4.3B and 4.4B) possesses two spiking modes, consistent with real data, but at shorter delays than found in the observed ISI histogram. Finally, the simulated spectra and observed spectra both possess similar low frequency peaks near 10 Hz, but dissimilar broad peaks at higher frequencies. The real spectra possess broad peaks centered near 100 Hz, but the simulated spectra possess broad peaks centered near 250 Hz, which is consistent with the fact that the simulated ISI histograms show a faster spiking mode than the observed ISI histograms (in Figure 4.3C and 4.4C). The differences in the fast spiking activity between the model and observed spike time data likely reflect limitations in the biophysical model used. In order to capture exact features of the fast activity more accurately, we may require a model with additional intrinsic currents or more complicated structure (e.g., a multicompartmental model). On the whole, small discrepancies distinguish the descriptive statistics of the real and simulated data. Yet, the estimated biophysical model, consisting of only a single compartment and 3 dynamic currents, still generates spike trains similar to the observed data in terms of the distribution of the inter-spike intervals and the point process spectrum.

## Discussion

Connecting real-world data with sophisticated computational models is a fundamental issue in modern science. Here, we have extended a method we previously presented [21] to link observed neural spike time data with a conductance based computational model. An initial descriptive and statistical analysis of the spike time data observed in four IB cells revealed two classes of behavior: regular spiking activity and bursting. To characterize the mechanisms of these behaviors, we constructed a biophysical model and estimated parameters of an unknown “mystery current” in this model using the SMC method. According to the estimates of the parameters, the two classes of spiking activity derive from the same type of intrinsic current: a slow, depolarization activated, hyperpolarizing current, consistent with the known M-current in the IB cell. Different biophysical features – the drive current and maximum conductance of the mystery current – explain the two different classes of behavior. By combining the observed spike time data with the computational model, the SMC method suggests the specific biophysical mechanisms producing the observed activity and identifies the regions of the 6-dimension parameter space capable of reproducing the observed data. We note that these two classes of behavior may represent states within a continuum of dynamics. In this case, with additional data, we expect the particle filtering approach to reveal the biophysical model parameters supporting such a continuum.

We note that the simulated ISI distributions estimated from the biophysical model possess some inconsistencies with the real ISI distribution (Fig. 4). For the two bursting cells, the simulated spiking is faster than the observed spiking. In general, such inconsistencies may result from model misspecification, which may occur in multiple ways. For example, the model may lack an intrinsic current or additional compartments whose inclusion would better fit the data. Biologically, the reduced high frequency firing observed *in vitro* may result from failures in back-propagation of axonal spikes into the large-capacitance somatic compartment; a more accurate model could include a multi-compartment geometry. Alternatively, direct recordings near the axon may alleviate this issue, although such recordings are experimentally difficult. In general, all computational models are misspecified, and can always be modified to incorporate further biological realism. However, even the single compartment model implemented here provides biological insight. This model successfully captures the essential features of the observed neuronal data, without representing a true generative model of the data. Given only the spike time data, the proposed model suggests the type of slow current known to play an important role in these cells. Therefore, the value of this model is the successful identification of an unknown ionic current species vital to the cell dynamics although the model does not capture all biophysics of the cell or changes to the biological system inherent in the experimental recording process.

The proposed approach to parameter estimation, although successful in this case, is limited in two important ways. First, the approach requires some knowledge of the underlying equations that govern the neuronal dynamics. In this case, we knew that an intrinsic current paced the observed activity, and therefore developed a model to exploit this knowledge. In general, model development will be more successful when supported by knowledge of the features to be studied. A model inconsistent with the neuronal system under investigation will lead to inaccurate biophysical conclusions, even if the parameter estimation converges. However, because the model is biophysical, the resulting estimates are testable in experiments. Through interactions between this parameter estimation procedure and experiments, an inaccurate model can be refuted experimentally and a more accurate model proposed. In this work, the parameter estimation results were compared to the known biophysical mechanism pacing the observed activity (an M-current) and found to be consistent. Second, the model was limited to a single compartment cell, and a limited number of the parameters were estimated. As computational resources continue to improve, estimation will become more feasible for larger, more biophysically realistic models of single cells, and networks of interacting cells.

As computational resources improve, we propose that a closed-loop analysis will become possible, in which the SMC method combined with computational models can be used to propose the existence of possible candidate currents in real time from observed spike train data. The proposed candidate currents can then be tested in pharmacological experiments. In this way, the SMC method identifies candidate biophysical mechanisms that are experimentally testable, potentially reducing dramatically the numbers of experiments required to identify unknown mechanisms. Such an approach will become increasingly vital as high density recordings and observations from many simultaneous neurons become more common. We note that the SMC method easily extends to include network models of interacting neurons.

The approach in this paper outlines a general strategy for a practical data analysis paradigm of spike train data. Both statistical modeling and biophysical modeling characterize neuronal spike train data, but from different points of view, and these two approaches are typically applied independently. The proposed SMC method goes beyond standard analysis and modeling approaches by combing statistical and biophysical methods: the statistical analysis guides the biophysical modeling and the biophysical modeling lends mechanistic features to the statistical analysis. The resulting technique connects spike train data directly to a biophysical model and provides a principled link between the fields of experimental, statistical, and computational neuroscience.

## Methods

Our goal is, given a list of the spike times produced by a neuron, to identify biophysical mechanisms that could support the observed spiking activity. To do so, we use the observed spike times to constrain the parameters in a biophysical model of neural activity [21]. Briefly, this technique utilizes a sequential Monte Carlo (SMC) method, which incorporates biophysical modeling and point process theory into a state space framework. As we will show, this analysis links the observed spiking activity directly to specific biophysical mechanisms that are not immediately observable. To apply this SMC method to the observed neural spike times of interest here, we must construct a biophysical model capable of reproducing the observed spike train dynamics. To that end, we first perform visual data analysis and statistical modeling of the spike train data to characterize the spiking activity. The inferences arising from these analyses inform the biophysical model to which we apply the SMC method to estimate model parameters and dynamic variables, and draw inferences about the biophysical mechanisms generating the observed spike times.

### Data collection

Horizontal slices (450-μm thick) were prepared from adult male Wistar rats (150–250 g). Neocortical slices containing auditory areas and secondary somatosensory cortical areas were maintained at 34 C at the interface between warm wetted 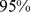 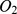 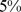 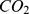 and artificial cerebrospinal fluid (aCSF) containing 3 mM KCl, 1.25 mM 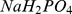, 1 mM 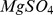, 1.2 mM 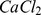, 24 mM 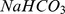, 10 mM glucose, and 126 mM 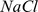. Extracellular recordings from secondary somatosensory cortex were obtained by using glass micropipettes containing the above aCSF (resistance 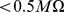). Intracellular recordings were taken with sharp microelectrodes filled with potassium acetate (resistance 30–90 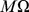). Signals were analog filtered at 2 kHz and digitized at 10 kHz. All neuronal recordings illustrated were taken from layer V. Neurons are shown to be intrinsically bursting by prior step-wise injection of depolarizing current through the recording electrode. Experimental conditions included the addition of 400 nM kainate to the bathing medium to generate a stable, persistent beta2 (20–30 Hz) rhythm visible in the local extracellular recordings. For additional details about the data collection, please see [26]. All procedures were conducted in accordance with the Animals (Scientific Procedures) Act 1986 (60/4313) and the University of York Policy on the Use of Animals in Scientific Research and approved by the Home Office (UK) Animals Scientific Procedures Department (ASPD).

### Descriptive statistics

Descriptive statistics provide a powerful and simple technique to characterize spike time data. Here we apply two visualizations of the spike time data: the inter-spike interval (ISI) histogram and the power spectrum. The ISI histogram presents the empirical distribution of ISIs. To compute the ISI histogram, we choose a bin size of 6 ms.

Next, we compute the power spectrum of the point process data to characterize the rhythmic features of the spiking. We use the multitaper framework [28]–[30] and choose the time-frequency product, 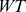, to preserve a frequency resolution near 1 Hz for 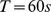. More specifically, we choose the time-frequency product to be 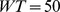 and make the standard choice for the number of tapers to be 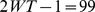. We compute confidence bounds using a jackknife method [31]. To implement these procedures, we utilized the “Chronux” package in MATLAB [32].

### Statistical modeling

As a second method to characterize the spike times, we consider a history-dependent statistical model of the data. To do so, we utilize a point process model by specifying a conditional intensity of spiking as a function past spiking activity. We first introduce notation for a discretized point process and second present a specific conditional intensity model that incorporates only the spiking history.

We choose a large integer 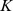 and partition the observation interval 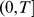 into 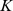 subintervals 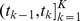 each of length 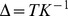. The integer 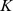 is chosen to be sufficiently large to guarantee that there is at most one spike per subinterval. Let 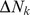 be the number of spikes counted in the time interval 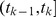.

A discretized point process can be completely characterized by its conditional intensity function 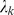 [10], [33] which defines the instantaneous probability of spiking at time 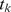 given the past spike history and other relevant covariates. Here, we construct a history-dependent conditional intensity model using cardinal spline functions of the past spike data [4]. We define 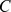 spline control points, extending 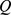 time steps into the past, 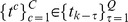, 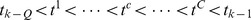. The history-dependent model is then constructed as follows,
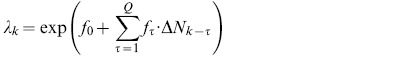
(1)

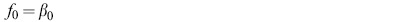


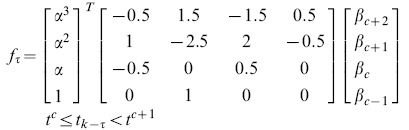
where 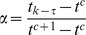 is the fractional distance of 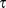 between neighboring spline control points, and 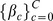 are the unknown parameters to estimate. The choice of the number and location of the spline control points depends on the shape of the function to approximate. It is not necessary to allocate many control points where the shape of the function does not change much. In our case, the function of interest describes the influence of the past spiking activity on the current spiking probability. During the refractory period the probability of spiking is close to zero, thus there is no need to assign many control points over this period. We choose the immediate past time step as the first control point. Then, depending on the refractory period computed through analysis of the ISI of the given data, we select the second control point immediately following the refractory period. Then, the remaining control points are picked evenly every 10 ms up to 200 ms (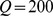).

We estimate the parameters, 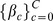, of the history-dependent model using maximum likelihood methods. Confidence intervals and p-values of the estimates are obtained by standard computations based on the observed Fisher information matrix [35]. To fit the spike times, we first discretize the continuous point process data with a discretization interval 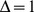 ms and then implement an iteratively reweighted least squares (IRLS) estimation algorithm in MATLAB using the package *glmfit*.

### Biophysical modeling

To relate the observed spike train data to (hidden) biophysical mechanisms, we estimate parameters in a computational model of neural spike train activity. We start with the Hodgkin-Huxley model [9], which describes three essential ionic currents (the sodium current, potassium current, and leak current) for spike generation in a mathematical manner. The dynamic interplay between these currents produces realistic behaviors, such as action potentials with refractory periods. As we show in the Results section, the observed spike train data exhibit intervals of bursting (i.e., repeated periods of rapid spiking and quiescence) not observed in the standard Hodgkin-Huxley model formulation. Therefore we begin with this standard biophysical model and then amend it to capture more types of spiking patterns (such as extended refractory periods and bursting activity). To do so, we augment the standard Hodgkin-Huxley model by including an additional “mystery current” with flexible dynamic features. In addition, we make the standard simplification that the activation variable of the sodium current is infinitely fast [34]. The dynamic equations for the biophysical model are as follows
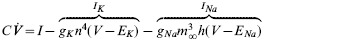
(2)

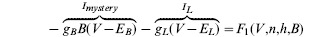


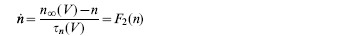


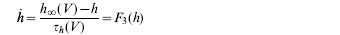


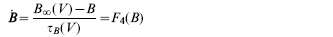
where



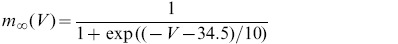
(3)

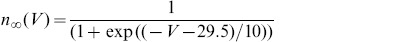


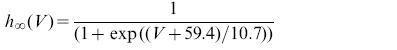


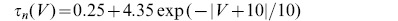


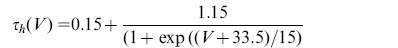


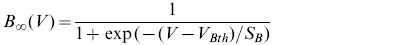


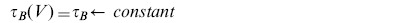



Notice that Eq. 2 is very similar to the original Hodgkin-Huxley model, but includes the new mystery current term 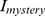. The mystery current has maximum conductance 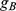, gating variable 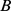, and reversal potential 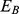. We assume here that the mystery current depends linearly on the gate 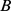, as is often the case for many intrinsic currents (see for example [36]). Nonlinear dependence is easily incorporated into the model, either as a fixed exponent of 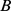 or as an unknown parameter to estimate. The mystery current dynamics evolve according to the steady state function 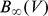, which depends on the voltage, and the fixed time constant 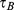 assumed to be independent of voltage.

For simplicity of the estimation problem, we consider only a single cell representation of the observed data. We note that the beta2 activity of interest here is known to depend on gap junction inputs from other cells, such that blocking gap junctions eliminates the beta2 activity [26]. However, the primary mechanism that sets the timescale of the beta2 activity in this system has been shown to be an intrinsic current (namely, an M-current) [26]. We therefore focus on this primary mechanism that paces the activity, and utilize a single cell representation of the intrinsically bursting cell motivated by a similar model in [37]. Parameter estimation for a complete network model, with many additional parameters, is of interest but beyond the scope of the current work. In addition, we assume that all currents except for the mystery current follow known kinetics as defined in [36]. Therefore the unknown parameters **Θ** are all associated with the mystery current 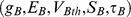 and the drive current 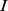 which controls the overall excitability of the cell; the goal of this work is to estimate these unknown parameters given only the observed spike times. All other parameters are fixed at the values in [36], 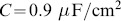, 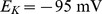, 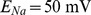, 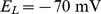, 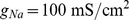, 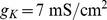, 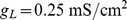.

### Combining point process theory and biophysical models

The statistical modeling (Section 2.3) and biophysical modeling (Section 2.4) methods provide two distinct approaches for characterizing the observed spike activity. Statistical spiking models are often used to describe simple relations between the spiking and other covariates (e.g., past spiking history), without describing the biophysical mechanisms that give rise to these relations. Biophysical models typically concentrate on the deterministic kinetics supporting qualitative features of the observed activity. In this section, we discuss a method linking these approaches by estimating biophysical model parameters from the observed spike train data through a point process framework. Our goal is to estimate the model parameters **Θ
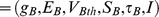
** based on the given spike times. We have recently proposed a recursive estimation method (a sequential Monte Carlo method) to solve this kind of problem [18]. We start by describing the biophysical dynamics using a state space framework. To do so, we discretize the continuous dynamic system (Eq. 2) with a discretization interval 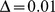 ms using Euler's method. The voltage (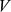) and gating variables (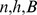) in discrete time are denoted as 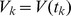, 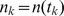, 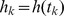, and 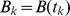 where 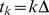. Then we define a state vector 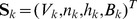. The continuous neural dynamics can be expressed in discrete time using a state-space model including a random noise term
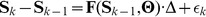
(4)where 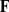 represents the function vector 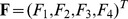 as defined in Eq. 2 and 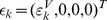, 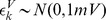.

We define a new conditional intensity 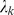, that depends on the unobserved subthreshold neural voltage trace, 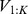, propagating through the constructed biophysical model with unknown parameter set 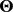 and evolution noise. We assume that 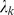 is a step function of the unobserved voltage trace 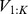,
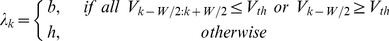
(5)where 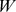 is the width of a window centered at 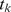, 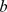 represents a baseline firing rate and 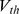 represents a voltage threshold determining the occurrence of a spike. This intensity function acts like a square wave, with every square having the same height (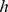), width (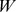) and baseline value (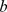).

For the biophysical model utilized here, we set 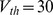 mV since the maximum voltage achieved during a spike is approximately 40 mV.

We chose 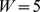 ms and 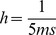 so that each square has (dimensionless) area 1, corresponding to an expected value of 1 spike. The parameter 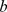 determines the baseline probability of spiking in the model at times when spikes in the data were not observed. Here, we choose 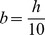, which means that we allow the biophysical model within the SMC procedure to produce simulated spikes away from observed spikes with 1/10 the probability of spiking within a window of length 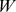 approximately centered at an observed spike.

Given the conditional intensity model (Eq. 5) for small 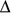, the probability of observing 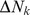 spikes at time 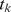 is
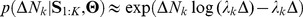
(6)Eq. 4 and Eq. 6 together form a state-space framework with spike observations (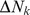). Given the observed spike times, we would now like to use this framework to estimate values for the state variables and the model parameters. The estimation algorithm is constructed using a sequential Monte Carlo (particle filtering) [38], [39] method, a technique which is widely used for estimation problems of high dimensional state space models. In the next subsection, we briefly describe the SMC algorithm; More details can be found in [21].

#### Estimation algorithm

The SMC algorithm (also known as a particle filter) is used to estimate the posterior probability distributions of the unknown quantities, given the observed spike times. Particle filters are so named because they represent the distribution of an unknown state using a collection of weighted samples, or particles. The initial samples of the unknown parameters are drawn from a uniform distribution over a large parameter space, which includes a variety of possible spike generation mechanisms. Here we initialized the particles of the parameter sets and variables as: 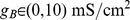, 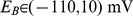, 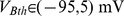, 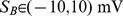, 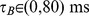, 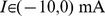; 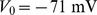, 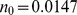, 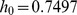, 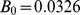. At any time step, each particle represents a set of possible values for the unknown variables and parameters, and the weighting function represents the probability associated with these values. As the number of particles becomes very large, this SMC characterization becomes more accurate. To balance the computational complexity and accuracy of the approximation, we use 10,000 particles. There are multiple approaches to computing the values and weights of each particle at any time. In this case, we construct a bootstrap particle filter [39], where the initial values for the particles at time 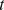 are sampled from the particles at the previous time step. The values of each particle are then updated by simulating Eq. 2. The weights of each particle are updated by multiplying by the likelihood of the observed spiking data at time 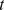 given by Eq. 6. Intuitively, each particle from the previous time step undergoes one step of the model dynamics. If the resulting state values are consistent with the newly observed data, the weight is enhanced. If the data are unlikely observed given the state values for a particle, its weight is reduced.

A common problem with particle filters is the degeneracy phenomenon, where after a few iterations, all but one particle will have negligible weight [38]. It has been shown that the variance of the weights can only increase over time, and thus, it is impossible to avoid the degeneracy phenomenon [38]. To reduce the effect of degeneracy, we use a resampling scheme. The basic idea of resampling is to eliminate particles that have small weights and to concentrate on particles with large weights. Here, we use a residual resampling scheme [40] at every spike time whereby particles with large weights are replicated based on their weight and particles with small weights have some probability of surviving and some probability of being eliminated. Let 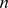 be the number of particles used and 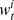 indicates the weight of the 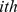 particle at time 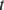. We retain 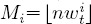 copies of the 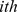 particle, where 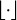 indicates rounding down to the nearest integer, and then obtain 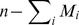 i.i.d. draws from the pool of particles with probabilities proportional to 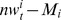, 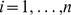. After resampling, the weights of each particle are reset to 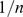. With more and more observations, the distribution of particles converges to the true posterior distribution. We construct estimates for the unknown quantities by computing their sample means over all particles, and construct approximate 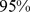 confidence intervals by computing the 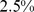 and 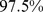 percentiles of the particle values. A pseudo-code description of the algorithm can be found in the Appendix of [21].

## References

[1] Shepherd G (2003) The synaptic organization of the brain. Oxford University Press, USA.

[2] Izhikevich E (2003) Simple model of spiking neurons. Neural Networks, IEEE Transactions on 14: 1569–1572. 10.1109/TNN.2003.820440 18244602

[3] Steriade M, McCormick D, Sejnowski T (1993) Thalamocortical oscillations in the sleeping and aroused brain. Science 262: 679–679. 8235588 10.1126/science.8235588

[4] Hodgkin A, Huxley A (1952) A quantitative description of membrane current and its application to conduction and excitation in nerve. The Journal of Physiology 117: 500. 12991237 10.1113/jphysiol.1952.sp004764 PMC1392413

[5] Prinz A, Billimoria C, Marder E (2003) Alternative to hand-tuning conductance-based models: construction and analysis of databases of model neurons. Journal of Neurophysiology 90: 3998–4015. 12944532 10.1152/jn.00641.2003

[6] Nadim F, OlsenØ, Schutter E, Calabrese R (1995) Modeling the leech heartbeat elemental oscillator I. interactions of intrinsic and synaptic currents. Journal of Computational Neuroscience 2: 215–235. 8521288 10.1007/BF00961435

[7] Traub R, Wong R, Miles R, Michelson H (1991) A model of a CA3 hippocampal pyramidal neuron incorporating voltage-clamp data on intrinsic conductances. Journal of Neurophysiology 66: 635–650. 1663538 10.1152/jn.1991.66.2.635

[8] Vanier M, Bower J (1999) A comparative survey of automated parameter-search methods for compartmental neural models. Journal of computational neuroscience 7: 149–171. 10515252 10.1023/a:1008972005316

[9] Paninski L, Pillow J, Lewi J (2007) Statistical models for neural encoding, decoding, and optimal stimulus design. Progress in Brain Research 165: 493–507. 17925266 10.1016/S0079-6123(06)65031-0

[10] Truccolo W, Eden U, Fellows M, Donoghue J, Brown E (2005) A point process framework for relating neural spiking activity to spiking history, neural ensemble, and extrinsic covariate effects. Journal of Neurophysiology 93: 1074–1089. 15356183 10.1152/jn.00697.2004

[11] Bhalla U, Bower J (1993) Exploring parameter space in detailed single neuron models: simulations of the mitral and granule cells of the olfactory bulb. Journal of Neurophysiology 69: 1948–1965. 7688798 10.1152/jn.1993.69.6.1948

[12] Druckmann S, Banitt Y, Gidon A, Schürmann F, Markram H, et al (2007) A novel multiple objective optimization framework for constraining conductance-based neuron models by experimental data. Frontiers in Neuroscience 1: 7. 18982116 10.3389/neuro.01.1.1.001.2007 PMC2570085

[13] Robinson P, Rennie C, Rowe D, O'Connor S (2004) Estimation of multiscale neurophysiologic parameters by electroencephalographic means. Human Brain Mapping 23: 53–72. 15281141 10.1002/hbm.20032 PMC6871818

[14] Huys QJM, Ahrens MB, Paninski L (2006) Efficient estimation of detailed single-neuron models. Journal of Neurophysiology 96: 872–90. 16624998 10.1152/jn.00079.2006

[15] Schiff S, Sauer T (2008) Kalman filter control of a model of spatiotemporal cortical dynamics. Journal of Neural Engineering 5: 1. 18310806 10.1088/1741-2560/5/1/001 PMC2276637

[16] Huys QJM, Paninski L (2009) Smoothing of, and parameter estimation from, noisy biophysical recordings. PLoS Comput Biol 5: e1000379. 19424506 10.1371/journal.pcbi.1000379 PMC2676511

[17] Ullah G, Schiff S (2010) Assimilating seizure dynamics. PLoS Comput Biol 6: e1000776. 20463875 10.1371/journal.pcbi.1000776 PMC2865517

[18] Lansky P, Ditlevsen S (2008) A review of the methods for signal estimation in stochastic diffusion leaky integrate-and-fire neuronal models. Biological cybernetics 99: 253–262. 18496710 10.1007/s00422-008-0237-x

[19] Mullowney P, Iyengar S (2008) Parameter estimation for a leaky integrate-and-fire neuronal model from ISI data. Journal of Computational Neurosciencee 24: 179–194. 10.1007/s10827-007-0047-5 17661164

[20] Paninski L, Pillow J, Simoncelli E (2004) Maximum likelihood estimation of a stochastic integrateand-fire neural encoding model. Neural Computation 16: 2533–2561. 15516273 10.1162/0899766042321797

[21] Meng L, Kramer M, Eden U (2011) A sequential Monte Carlo approach to estimate biophysical neural models from spikes. Journal of Neural Engineering 8: 065006. 22058277 10.1088/1741-2560/8/6/065006 PMC3529721

[22] Brown DA, Adams PR (1980) Muscarinic suppression of a novel voltage-sensitive K+ current in a vertebrate neurone. Nature 283: 673–6. 6965523 10.1038/283673a0

[23] Brown D (1988) M-currents: an update. Trends in Neurosciences 11: 294–9. 2465631 10.1016/0166-2236(88)90089-6

[24] McCormick DA, Wang Z, Huguenard J (1993) Neurotransmitter control of neocortical neuronal activity and excitability. Cereb Cortex 3: 387–98. 7903176 10.1093/cercor/3.5.387

[25] Vervaeke K, Gu N, Agdestein C, Hu H, Storm JF (2006) Kv7/KCNQ/M-channels in rat glutamatergic hippocampal axons and their role in regulation of excitability and transmitter release. J Physiol (Lond) 576: 235–56. 16840518 10.1113/jphysiol.2006.111336 PMC1995637

[26] Roopun A, Middleton S, Cunningham M, LeBeau F, Bibbig A, et al (2006) A beta2-frequency (20–30 Hz) oscillation in nonsynaptic networks of somatosensory cortex. Proceedings of the National Academy of Sciences 103: 15646–15650. 10.1073/pnas.0607443103 PMC1592532 17030821

[27] Wonnacott T, Wonnacott R (1990) Introductory statistics, volume 19690. Wiley Chichester.

[28] Thomson D (1982) Spectrum estimation and harmonic analysis. Proceedings of the IEEE 70: 1055–1096.

[29] Percival D, Walden A (1993) Spectral analysis for physical applications: multitaper and conventional univariate techniques, 583 Cambridge Univ Press, New York.

[30] Jarvis M, Mitra P (2001) Sampling properties of the spectrum and coherency of sequences of action potentials. Neural Computation 13: 717. 11255566 10.1162/089976601300014312

[31] Thomson D (2007) Jackknifing multitaper spectrum estimates. Signal Processing Magazine, IEEE 24: 20–30.

[32] Mitra P, Bokil H (2007) Observed brain dynamics. Oxford University Press, USA.

[33] Daley D, Vere-Jones D (2007) An introduction to the theory of point processes: volume II: general theory and structure, volume 2. Springer.

[34] Catmull E, Rom R (1974) A class of local interpolating splines. Computer Aided Geometric Design 74: 317–326.

[35] Pawitan Y (2001) In all likelihood: statistical modelling and inference using likelihood. OUP Oxford.

[36] Traub R, Buhl E, Gloveli T, Whittington M (2003) Fast rhythmic bursting can be induced in layer 2/3 cortical neurons by enhancing persistent na+ conductance or by blocking bk channels. Journal of neurophysiology 89: 909–921. 12574468 10.1152/jn.00573.2002

[37] Kramer MA, Roopun AK, Carracedo LM, Traub RD, Whittington MA, et al (2008) Rhythm generation through period concatenation in rat somatosensory cortex. PLoS Comput Biol 4: e1000169. 18773075 10.1371/journal.pcbi.1000169 PMC2518953

[38] Doucet A, De Freitas N, Gordon N (2001) Sequential Monte Carlo methods in practice, volume 1. Springer New York.

[39] Ergun A, Barbieri R, Eden U, Wilson M, Brown E (2007) Construction of point process adaptive filter algorithms for neural systems using sequential monte carlo methods. Biomedical Engineering, IEEE Transactions on 54: 419–428. 10.1109/TBME.2006.888821 17355053

[40] Liu J, West M (1999) Combined parameter and state estimation in simulation-based _ltering. Institute of Statistics and Decision Sciences, Duke University.

